# Factors Predicting Gaps in Access to Health-Promoting Resources Among Rural Older Adults

**DOI:** 10.1093/geroni/igaf122.2767

**Published:** 2025-12-31

**Authors:** Molly Wylie, Verena Cimarolli, Robyn Stone, Regan McManus, Ashley Washington

**Affiliations:** LeadingAge, Washington, District of Columbia, United States; LeadingAge, Washington, District of Columbia, United States; LeadingAge, Washington, District of Columbia, United States; Lutheran Services in America, Washington, District of Columbia, United States; Lutheran Services in America, Washington, District of Columbia, United States

## Abstract

Many older adults living in rural communities have limited access to resources that promote health. Yet, little is known about the factors contributing to disparities in access to specific resources. Therefore, this study identified sociodemographic predictors of gaps in access to five resources (transportation, social support, food security, healthcare, and safe housing) among rural older adults (N = 795). Data was collected as part of an evaluation of the Rural Aging Action Network (RAAN) - a national collaborative that mobilizes existing and recruits new community-based services and supports. Multivariate logistic regressions examined sociodemographic predictors of gaps in resource access among RAAN users. Income-restricted older adults were twice as likely to have gaps in safe housing (95% CI [1.16, 4.78]), transportation (95% CI [1.11, 4.09]), or healthcare (95% CI [1.20, 4.25]), over three times more likely to have a food security gap (95% CI [1.44, 7.39]), and almost four times as likely to have a social support gap (95% CI [1.85, 8.37]) compared to those with adequate income. Older adults of color were over twice as likely to have a transportation gap (95% CI [1.12, 6.14]) and over three times as likely to have a food security gap (95% CI [1.30, 8.28]) compared to Whites. Veterans were over twice as likely to have a housing gap (95% CI, [1.04, 6.57]), yet 81% less likely to have a social support gap (95% CI [0.04, 0.88]) compared to non-veterans. Study findings can help providers identify rural older adults with greatest service needs.

